# The Genome-Wide Identification of Long Non-Coding RNAs Involved in Floral Thermogenesis in *Nelumbo nucifera* Gaertn

**DOI:** 10.3390/ijms23094901

**Published:** 2022-04-28

**Authors:** Jing Jin, Yu Zou, Ying Wang, Yueyang Sun, Jing Peng, Yi Ding

**Affiliations:** 1 Key Laboratory of Plant Resource Conservation and Germplasm Innovation in Mountainous Region (Ministry of Education), College of Life Sciences, Guizhou University, Guiyang 550025, China; 2 State Key Laboratory of Hybrid Rice, Department of Genetics, College of Life Sciences, Wuhan University, Wuhan 430072, China; zouxiaoyu@whu.edu.cn (Y.Z.); yingwang@whu.edu.cn (Y.W.); yueyangsun@whu.edu.cn (Y.S.); 3 Institute of Vegetable, Wuhan Academy of Agricultural Science, Wuhan 430065, China; pengjing67@163.com

**Keywords:** lncRNA, floral thermogenesis, *Nelumbo nucifera*, strand-specific RNA sequencing, endogenous target mimics, WGCNA

## Abstract

The sacred lotus (*Nelumbo nucifera* Gaertn.) can maintain a stable floral chamber temperature when blooming, despite ambient temperature fluctuations; however, the long non-coding RNAs (lncRNAs) involved in floral thermogenesis remain unclear. In the present study, we obtain comprehensive lncRNAs expression profiles from receptacles at five developmental stages by strand-specific RNA sequencing to reveal the lncRNAs regulatory mechanism of the floral thermogenesis of *N. nucifera*. A total of 22,693 transcripts were identified as lncRNAs, of which approximately 44.78% had stage-specific expression patterns. Subsequently, we identified 2579 differential expressed lncRNAs (DELs) regulating 2367 protein-coding genes mainly involved in receptacle development and reproductive process. Then, lncRNAs with floral thermogenesis identified by weighted gene co-expression network analysis (WGCNA) were mainly related to sulfur metabolism and mitochondrial electron transport chains. Meanwhile, 70 lncRNAs were predicted to act as endogenous target mimics (eTMs) for 29 miRNAs and participate in the regulation of 16 floral thermogenesis-related genes. Our dual luciferase reporter assays indicated that lncRNA LTCONS_00068702 acted as eTMs for miR164a_4 to regulate the expression of *TrxL2* gene. These results deepen our understanding of the regulation mechanism of floral thermogenesis by lncRNAs and accumulate data for further research.

## 1. Introduction

The rapid development and wide application of high-throughput sequencing technology has led to the discovery of a large number of non-coding RNAs. Transcriptome studies of multiple species have revealed that more than 90% of the genomes produced non-coding RNA [1,2]. Non-coding RNAs can be classified according to their length: small RNAs, medium-sized ncRNAs, and long ncRNAs [3]. Long non-coding RNA (lncRNA) is more than 200 nt in length and lacks protein-coding potential [4]. They can be divided into intergenic ncRNAs, intronic ncRNAs, natural antisense transcripts (NATs) based on their origins of the genome and transcribed by RNA polymerase II or III in most eukaryotes, polymerases IV/V in plant [5,6,7,8]. LncRNAs can be located in the nucleus or cytoplasm with poor conservation and participate in biological activities on multiple regulatory levels, such as transcriptional, translational, post-translational, and post-transcriptional levels [9,10]. Although the importance of lncRNAs has been recognized in recent years, research in the plant field lags far behind the animal field.

The first identified lncRNA in plants was *Enod40*, which was expressed during nodular organogenesis in the *Medicago* plant and related to growth control and differentiation [11]. Subsequently, next-generation sequencing technology enables a large number of lncRNAs to be identified in model plants and non-model plants, such as *Arabidopsis*, rice, peach, and *Brassica rapa* [12,13,14,15]. Functional studies showed that plant lncRNA could regulate the development of reproductive organs and respond to abiotic and biotic stresses. For example, *BcMF11* is a pollen-specific expressed lncRNA in Brassica campestris. Antisense RNA transgenic plants of *BcMF11* showed obvious morphological defects and abnormal pollen development, which indicated the vital role in the pollen development process [16]. Rice lncRNA XLOC_057324 is specifically expressed in young panicles and ovules. T-DNA insertion mutant plants indicated that the reduced abundance of XLOC_057324 led to an earlier flowering time than the wild type, which reveals that XLOC_057324 plays a role in young panicle development [15]. The long intergenic non-coding RNA *LINCAP2* is close to the *APETALA2* (*AP2*) gene, which is related to the floral structure. The expressions of *LINCAP2* and *AP2* are negatively correlated in *Arabidopsis* infected with turnip crinkle virus (TCV). Overexpressed *LINC-AP2* transgenic plants have lower *AP2* gene expression and aberrant flower structure in *Arabidopsis* [17]. In addition, the floral development is extremely sensitive to external stress, which may lead to sterility [18]. Some lncRNAs may become potential balance factors for plant growth under different environmental conditions. They can not only participate in plant reproduction, but can also be regulated by the external environment. The most representative lncRNAs are cold-induced COLDAIR and COOLAIR, which promote flowering by regulating the *FLOWERING LOCUS C* (*FLC*) gene [3]. *FLC* encodes a transcription factor containing the MADS domain and directly inhibits the flowering-related promoters SOC1, FT, and FD [19]. Both COLDAIR and COOLAIR are transcribed from *FLC* and inhibit the expression of *FLC* via the function in histone modification [20,21].

Although the genome-wide identification and functional analysis of lncRNA have been carried out in some plants, the information of lncRNA in *N. nucifera* was still poorly understood. *N. nucifera* is an important aquatic horticultural plant with nutritional, ornamental, and medicinal values [22]. Therefore, increasing attention has been gained from the scientific community for it. Unlike other aquatic plants, *N. nucifera* has several unique characteristics, especially floral thermogenesis [22]. The floral thermogenesis phenomenon showed that the temperature of the floral chamber could be maintained at 30–35 °C without changing with the external environment during the flowering process in *N. nucifera* [23]. It can provide a warm environment for the floral chamber to ensure the normal development of flower organs. Sufficient heat return can also attract flower-visiting insects to promote pollination [24]. Overall, these phenomenon facilities the reproductive success of flowering plants. To date, there is no consolidated explanation for the regulation mechanism of floral thermogenesis in academia. Previous research has mainly focused on enzymes that regulate heat production in plants, such as AOX and UCP. In *N. nucifera,* oxygen isotope discrimination techniques were used to measure the alternative pathway flux and cytochrome pathway flux of receptacles [25]. The results indicate that AOX, rather than UCP, plays an important role in the process of floral thermogenesis [25]. In the respiratory chain, electrons are directly transferred to molecular oxygen through alternating oxidase without passing through the cytochrome system. Therefore, the synthesis of ATP decreased and the released heat energy increased [26,27]. Moreover, the floral thermogenesis phenomenon was also related to epigenetic regulation, and miRNAs have been reported to be involved in this process by targeting genes associated with cellular respiration and the energy metabolism pathway [28,29]. Numerous studies have also shown that lncRNAs could not only regulate flower processes, but also respond to extreme temperature environments [30,31]. However, the role of lncRNAs in floral thermogenesis is still ambiguous.

In the present study, a total of 15 strand-specific sequencing libraries are constructed using receptacles from five different developmental stages in *N. nucifera*. The expression profiles of lncRNAs during the receptacles’ development processes were obtained by bioinformatics analysis. Then, the floral thermogenesis-related lncRNAs are screened out based on their expression patterns by weighted gene co-expression network analysis (WGCNA), and the regulatory roles of these lncRNAs are also revealed by functional analysis. Subsequently, the predicted lncRNA–miRNA–mRNA interaction network during the floral thermogenesis process is confirmed by dual-luciferase reporter assays. These results deepen our perspective of lncRNAs in lotus species, which are essential for flower development and floral thermogenesis, and also contribute clues to reveal the regulatory mechanism of floral thermogenesis.

## 2. Results

### 2.1. The Identification and Characterization of lncRNAs in the Floral Thermogenesis of N. nucifera

**Figure 1:**
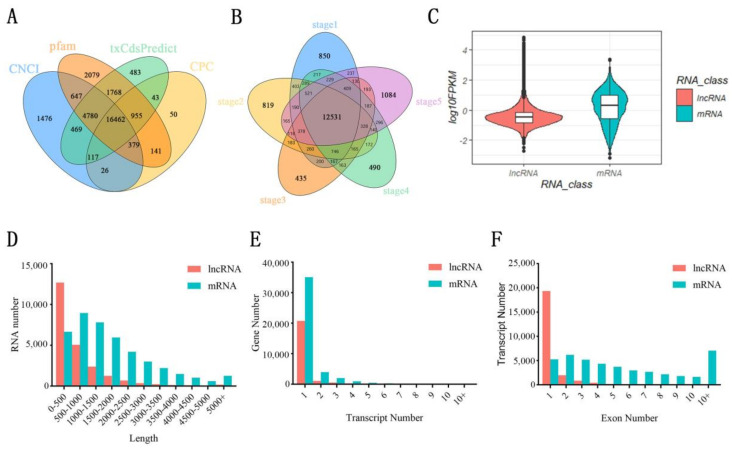
Characteristics of the identified transcripts. (**A**) Statistical results of lncRNA predicted by four different methods. (**B**) Distribution of lncRNAs in the receptacle at different developmental stages. (**C**) The expression levels of mRNA and lncRNA. (**D**) Statistical results of mRNA and lncRNA lengths. (**E**) Statistical results of mRNA and lncRNA transcript numbers. (**F**) Statistical results of mRNA and lncRNA exon numbers. CPC, coding potential calculator; CNCI, coding-non-coding index; Pfam, protein families database.

In order to elucidate the role of lncRNAs in floral thermogenesis, receptacles at five different developmental stages were used as sequencing materials in *N. nucifera*. A total of 15 strand-specific RNA libraries were constructed for sequencing on Illumina HiSeq platform. Each sample produced an average of 12.60 Gb of data, and the average genomic alignment rate was 67.50% (Table S1). Three predictive types of software (CPC, txCdsPredict, and CNCI) and one database (pfam) were used to predict the coding capability of new transcripts (Figure 1A). Only when at least three of the four methods were consistent, did we confirm that the transcript was either mRNA or lncRNA. As a result, a total of 22,693 transcripts were identified as lncRNAs. Among them, 12,531 lncRNAs (55.22%) were distributed in five developmental stages without period specificity (Figure 1B). Meanwhile, a large number of lncRNAs were identified, only at certain stages. For example, the number of specifically expressed lncRNAs was the highest at stage 5 and the lowest at stage 3, which was 1084 and 435, respectively (Figure 1B). In addition, a total of 37,019 transcripts were identified as mRNAs. Overall, the mRNA expression level and transcriptome length of these samples were significantly higher than that of lncRNAs. The length of majority lncRNAs ranges from 200 to 500 nt, while most mRNAs are above 500 nt in length (Figure 1C,D). Meanwhile, the transcript number and exon number of mRNAs are also significantly higher than that of lncRNAs. The majority of lncRNAs have only one transcript and one exon number (Figure 1E,F). Part of the mRNAs contained more exons and transcript numbers because of alternative splicing.

### 2.2. Differentially Expressed lncRNAs during the Receptacle Development Process

**Figure 2:**
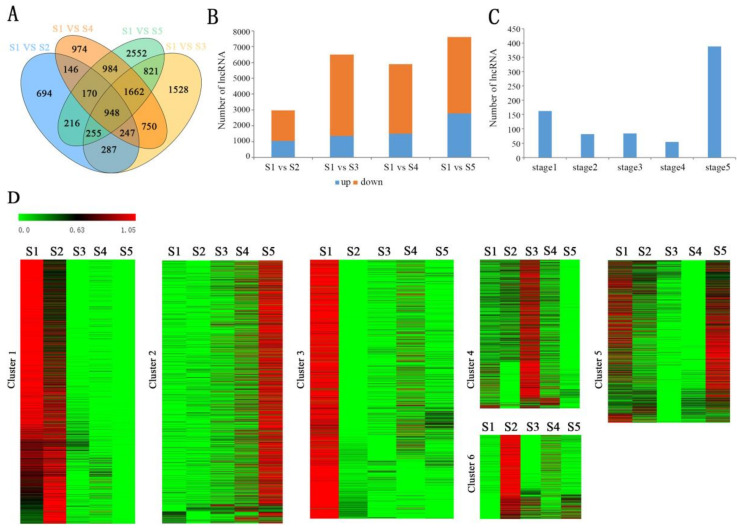
Identification and analysis of differentially expressed lncRNAs. (**A**) The distribution of DELs in each comparison group. (**B**) Statistical results of the up-regulated or down-regulated DEL numbers in each control group. (**C**) The statistical results of the stage-specific DEL numbers. (**D**) K-means clustering results of DELs.

The filtering conditions of significant differential expressed lncRNAs (DELs) were fold change ≥ 2.00 and adjusted *p*-value ≤ 0.001. Finally, a total of 2963, 6508, 5891, and 7608 DELs were screened out when S1 vs. S2, S1 vs. S3, S1 vs. S4, and S1 vs. S5, respectively. Meanwhile, 247 DELs were commonly shared in S1 vs. S2, S1 vs. S3, and S1 vs. S4, which were differentially expressed between the pre-thermogenesis and thermogenesis stages in receptacle (Figure 2A). Moreover, the number of down-regulated lncRNAs was significantly higher than that of up-regulated lncRNAs among all the DELs (Figure 2B). For example, 6508 lncRNAs were differentially expressed between stages 1 and 3, of which 1340 (20.5%) were up-regulated and 5168 (79.4%) were down-regulated in stage 3 (Figure 2B). The stage-specific DELs were also distributing inhomogeneity during the receptacle development process. The receptacle of non-thermogenesis periods (stages 1 and 5) contained more stage-specific expression lncRNA than that in thermogenesis periods (stages 2, 3, and 4) (Figure 2C). In addition, K-mean clustering was conducted in order to elaborate the expression patterns of these DELs in more detail. A total of 6 clusters were obtained using MeV software, and lncRNAs in different clusters had different expression patterns (Figure 2D). Furthermore, these DELs exhibited significant period-specific expression patterns. For instance, the lncRNAs in cluster 1 were highly expressed in the first two stages of the receptacle development process. The lncRNAs in clusters 2, 3, 4, and 6 existed with specific high expression levels in stages 5, 1, 3, and 2, respectively. Meanwhile, the lncRNAs in cluster 5 were higher expressed during the non-thermogenic period of the receptacle (stages 1 and 5) (Figure 2D).

**Figure 3:**
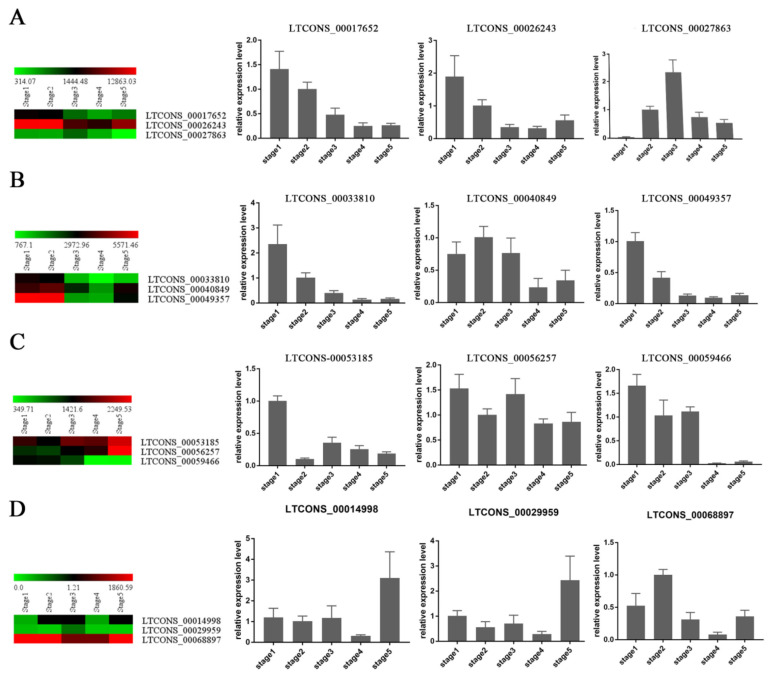
The expression patterns of 12 randomly selected DELs were verified by qRT-PCR. The heatmaps represent the transcription expression (FPKM) and the column diagram represents the relative expression levels. (**A**) The expression patterns of LTCONS_00017652, LTCONS_00026243, and LTCONS_27863. (**B**) The expression patterns of LTCONS_00033810, LTCONS_00040849, and LTCONS_00049357. (**C**) The expression patterns of LTCONS_00053185, LTCONS_00056257, and LTCONS_00059466. (**D**) The expression patterns of LTCONS_00014998, LTCONS_00029959, and LTCONS_00068897. Bar = means +/− SD from three biological repeats.

In addition, we randomly selected 12 DELs for qRT-PCR to verify their expression patterns. The qRT-PCR results of most DELs showed the same tendency as the RNA-seq results, which indicate that the expression pattern of lncRNA based on sequencing data is reliable (Figure 3). Period-specific expression patterns indicate that these DELs may play an important role in the development of receptacles.

### 2.3. The Identification and Functional Analysis of Target Genes of DELs

**Figure 4:**
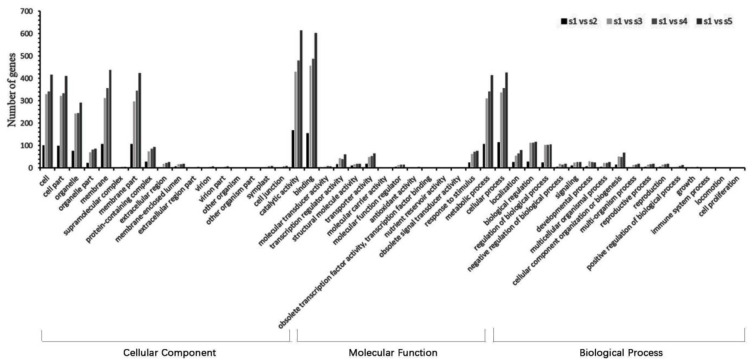
GO annotation results of DEL cis-target genes in different comparison groups.

The function of lncRNAs can be realized by cis acting on target genes. The lncRNAs located within 10 kb upstream or 20 kb downstream of protein-coding genes on the same chromosome are searched, as the cis-acting mode and the adjacent mRNA is screened out as its target gene. A total of 2579 DELs may regulate 2367 protein-coding genes through the cis-acting mode (Table S2). Approximately 306 lncRNAs can regulate multiple target genes and 421 mRNAs were cis-regulated by at least two lncRNAs. To further comprehend the function of these DELs, GO annotations were performed on their target genes. A total of 17,12, and 19 GO terms were contained in the cellular component, molecular function, and biological processes (Figure 4). The most enriched GO terms were the membrane, binding, and cellular process, respectively. Moreover, a large number of target genes were annotated in GO terms as a response to stimulus, developmental process, reproductive process, and growth, which indicated the corresponding lncRNAs of these target genes might participate in the receptacle development process and reproductive process of *N. nucifera* (Figure 4).

### 2.4. Screening of the lncRNAs and Genes Related to Floral Thermogenesis

**Figure 5:**
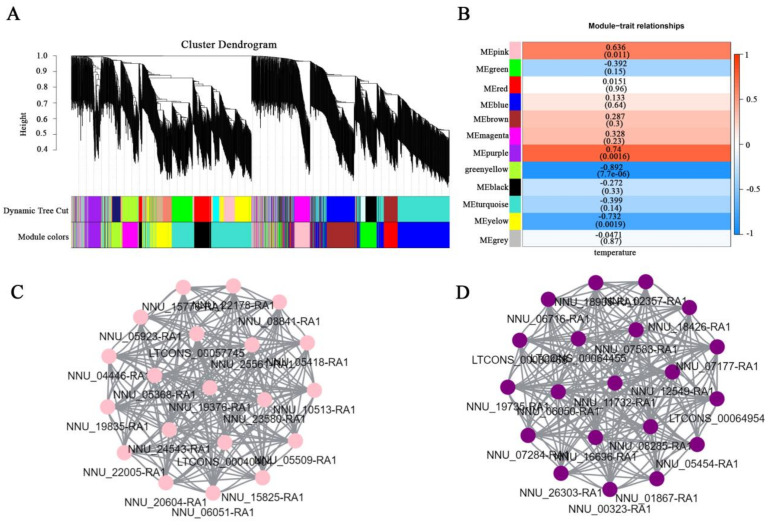
Floral thermogenesis-related transcripts were identified by WGCNA. (**A**) Hierarchical cluster tree showing co-expression modules. (**B**) Correlation analysis between modules and traits. (**C**) Hub genes in the MEpink module. (**D**) Hub genes in the MEpurple module. ME, module eigengene.

In order to further reveal the role of lncRNAs in floral thermogenesis, weighted correlation network analysis (WGCNA) was performed. In this study, a total of 12 co-expression modules with different colors were constructed (Figure 5A). Genes in the same module have a high correlation coefficient with each other. Meanwhile, the temperature change trend of the receptacle was regarded as phenotypic information during the floral thermogenesis process in *N. nucifera*. The correlation between gene modules and phenotypes was calculated to identify the floral thermogenesis-related module (Figure 5B). Among them, the module–trait relationships showed that MEpink and MEpurple modules were significantly positively correlated with traits, and MEyellow and greenyellow modules were significantly negatively correlated with traits (Figure 5B). The MEpink module has 97 members, including 25 lncRNA and 72 protein-coding genes. The MEpurple module has 87 members, including 20 lncRNAs and 67 protein-coding genes (Table S3). The members of the MEpink and MEpurple modules had higher expression levels in the thermogenic period (stages 2–4) than in the non-thermogenic period (stages 1 and 5) (Figure S1). Hub genes in these two modules were screened out to understand the co-expression networks in more detail (Figure 5C,D). Alternative oxidase 1a (NNU_06050) and alternative oxidase 1b (NNU_06051), which have been identified as floral thermogenesis-related genes, were included in the hub genes of the MEpurple and MEpink modules, respectively (Figure 5C,D). Moreover, the GO-enrichment analysis of the mRNAs was also performed to reveal the function of these two modules. The most significant enrichment biological process in the MEpurple module was sulfate absorption (Figure S2). Meanwhile, the mRNAs of the MEpink module were enriched in “Mitochondrial electron transport, ubiquinone to cytochrome C”, which was considered to be the main route of floral thermogenesis (Figure S3). Therefore, lncRNAs in the MEpink and MEpurple modules may play an important role in floral thermogenesis in *N. nucifera*.

### 2.5. LncRNA Can Regulate Transcript Expression by Acting as eTMs for miRNA

**Figure 6:**
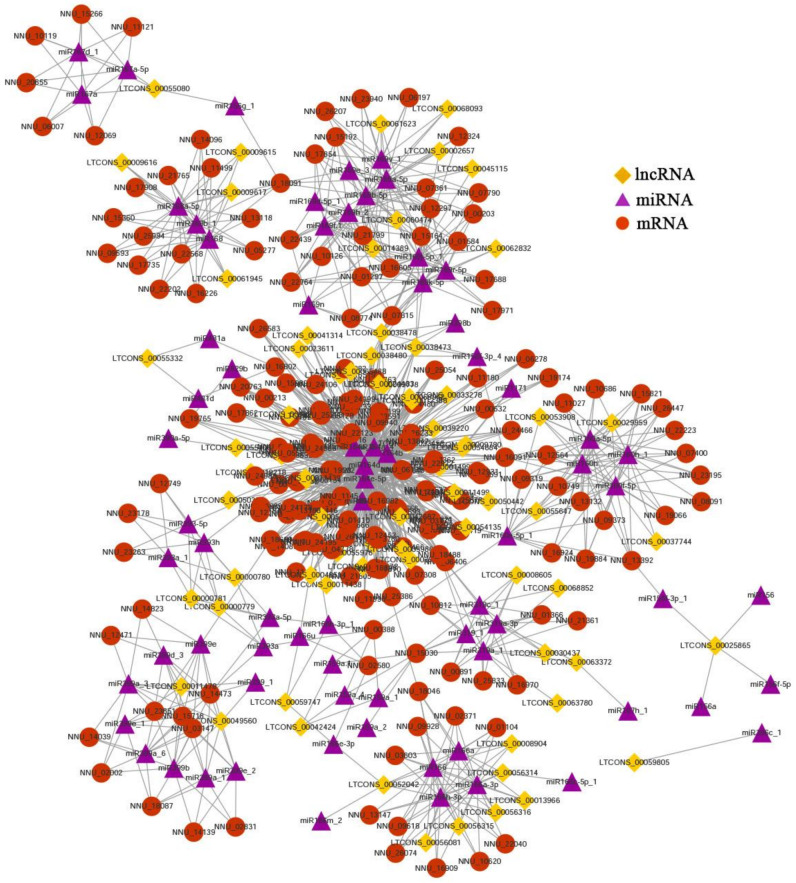
The mRNA–miRNA–DEL interaction network during the receptacle development process. The diamond-shaped, triangular, and round nodes represent DE-lncRNAs, miRNAs, and mRNAs, respectively.

LncRNA can act as the endogenous target mimics (eTMs) for miRNAs and compete with mRNAs to relieve their degradation by miRNAs. To further clarify the regulatory relationship between miRNA, mRNA, and lncRNA during the floral thermogenesis process, the miRanda algorithm was used to predict the interaction between 289 miRNAs and 9415 transcripts in the present study. A total of 75 DELs were predicted as eTMs for miRNA (Table S4). Meanwhile, 182 known mRNAs and 73 known miRNAs were also contained in the lncRNA–miRNA–mRNA interaction network (Figure 6). A large number of known miRNAs are associated with temperature response and the flower development process, such as miR159, miRNA160, miR164, miR169, miR166, miRNA167, and miR319. In the interaction network, the majority of lncRNAs have a weaker ability to bind corresponding miRNA than those of mRNAs. For example, the prediction results indicate that miR160a-5p has 19 targets, including 3 lncRNAs and 16 mRNAs. MiR168 has 18 targets, including 4 lncRNAs and 14 mRNAs (Table S4). The number of mRNAs binding to the same miRNA was more than that of lncRNA. In addition, several different miRNAs can simultaneously regulate the same target. For example, miR168, miR168a-5p, and miR168b_1 can synchronously regulate the expression of LTCONS_00061945, LTCONS_0009617, LTCONS_0009616, LTCONS_0009615, and NNU_05277 (Figure 6). The analysis of genes involved in the lncRNA–miRNA–mRNA interaction network revealed that 16 mRNAs related to floral thermogenesis could compete with 70 lncRNAs for binding 29 miRNAs (Table S4). Moreover, the number of mRNAs binding to the same miRNA was less than that of lncRNA. For example, 7 transcripts were predicted as the targets of miR169a-5p, including 6 lncRNAs and 1 mRNA (NNU_07790-RA).

**Figure 7:**
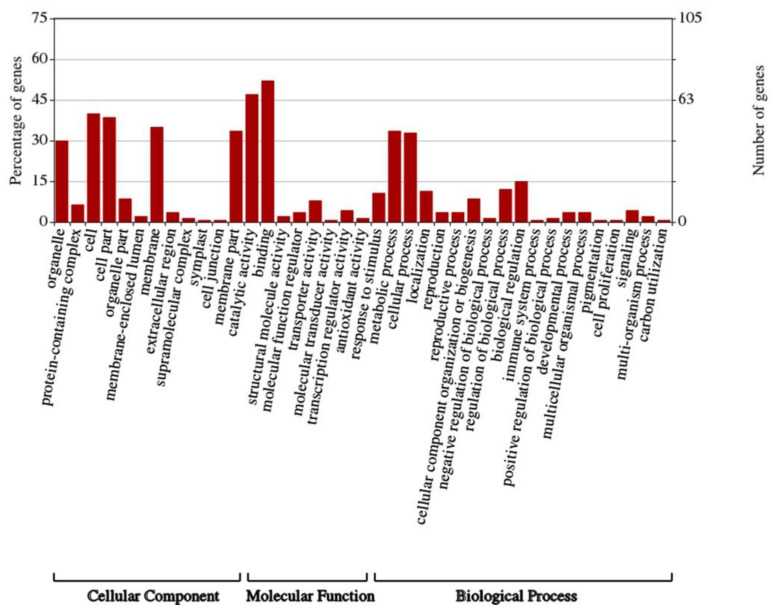
Go annotation results of mRNAs in the ceRNA network.

Subsequently, GO annotated analysis was performed on mRNAs by WEGO to reveal the function of the interaction network (Figure 7). In the molecular function, some genes were annotated as “redox activity” (GO:0016491). Several mRNAs were annotated as “antioxidant activity” (GO:0016209) and “peroxidase activity” (GO:0004601). In the biological process, a large number of genes were annotated as GO terms associated with the response to stimuli, including “response to biological stimuli “(GO:0009607), “respond to abiotic stimuli” (GO:0009628), and “response to external stimuli” (GO:0009605). Meanwhile, a few genes have been annotated as GO terms related to reproductive processes, including “pollen recognition” (GO:0048544), “interactions between pollen and pistil” (GO:0009875), and “pollination” (GO:0009856) (Table S5). These results suggest that lncRNAs might play a vital role in flower development and floral thermogenesis through the lncRNA–miRNA–mRNA interaction network.

### 2.6. Experimental Validation of LTCONS_00068702 as eTMs for miR164a_4

**Figure 8:**
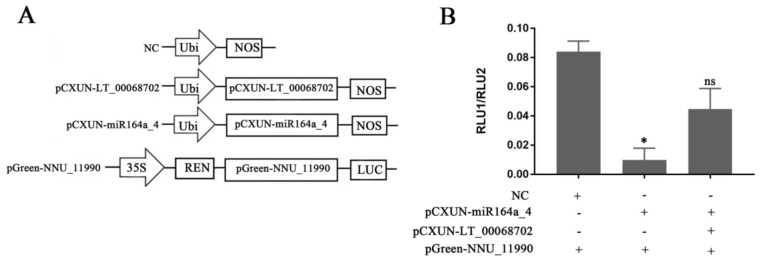
Dual-luciferase reporter assay. (**A**) Schematic diagram of pCXUN and pGreenII-0800-LUC vector. (**B**) The interaction results of LTCONS_00068702-miR164A_4-NNU_11990 by the double-luciferase reporter gene assay. * Represents the significant difference between the experimental and control groups using one-way ANOVA (* *p* value < 0.05); ns stands for “not significant”. NOS, terminator; Ubi, ubiquitin promoter; REN, Renilla luciferase; LUC, firefly luciferase.

According to the predicted lncRNA–miRNA–mRNA interaction network in *N. nucifera*, LTCONS_00068702 can compete with NNU_11990 for binding miR164a_4 (Figure 6). NNU_11990 encodes thioredoxin-like protein 2 (*TrxL2*), which is involved in intracellular redox reactions. The expression pattern of NNU_11990 was similar to the trend of receptacle temperature change, which was higher during the thermogenesis stages. Therefore, NNU_11990 may play a role in floral thermogenesis in *N. nucifera*. To verify the interactive relationship of LTCONS_00068702-miR164A_4-NNU_11990, we performed the dual-luciferase reporter assay using pGreenII 0800-LUC vector (Figure 8). The experimental results indicate that the ratio of firefly fluorescence (RLU1) to Renilla fluorescence (RLU2) is significantly decreased in lotus protoplasts after co-transfection with miR164a_4 and NNU_11990, compared to the negative control. The ratio of RLU1 to RLU2 is not significantly different from that of the control group after co-transfection with LTCONS_00068702, miR164a_4, and NNU_11990 (Figure 8). These results indicate that LTCONS_00068702 could act as eTMs and compete with NNU_11990 for binding miR164a_4.

## 3. Discussion

### 3.1. LncRNAs with Stage-Specific Expression Patterns during the Receptacle Development Process

The floral thermogenesis phenomenon is critical for the reproductive success of *N. nucifera* [32,33,34]. In order to understand the lncRNA regulatory mechanism of floral thermogenesis, we performed strand-specific library sequencing using receptacles from five different developmental stages. The sequencing results show that about 55.22% of the lncRNAs are distributed during the whole developmental stages, and the other lncRNAs show stage-specific expression patterns. Moreover, the number of specifically expressed lncRNAs was the largest at stage 5 and the least at stage 3, which was 1084 and 435, respectively (Figure 1). This phenomenon may be associated with the functional transition of the receptacle. The receptacle is the main thermogenic tissue in *N. nucifera* and has the maximum heat production at stage 3 [35,36]. Previous studies have shown that floral thermogenesis is related to plant cyanide-resistant respiration. In the respiratory chain, electrons are directly transferred to molecular oxygen through alternating oxidase without passing through the cytochrome system. Therefore, the synthesis of ATP decreased and the released heat energy increased [35,36]. Decreased ATP synthesis may limit certain biological activities that require energy. The number of lncRNAs involved in the corresponding biological process also decreased. In stage 5, the petals and stamens fall off from the receptacle that ended the thermogenesis activity. The color of the receptacle gradually changes from yellow to green and the function of the receptacle changes from a heat-generating organ to a tphotosynthetic organ [36]. The functional transformation of he receptacle involves complex regulatory networks, so the number of lncRNAs specifically expressed in the receptacle was the largest in this stage. In addition, DELs were identified to better understand the role of lncRNAs during the receptacle development process. A large number of DELs have been identified, of which down-regulated DELs were more than up-regulated DELs (Figure 2). The results might indicate that cell metabolism is vigorous in the early stage of the receptacle and highly expressed lncRNAs were required to participate in biological regulation, which benefited to provide a substance basis for subsequent growth and development. Meanwhile, the expression patterns of DELs were also investigated during the receptacle development process by K-means clustering. The results show that lncRNAs in cluster 1 are highly expressed in the first two stages. The lncRNAs in clusters 2, 3, 4, and 6 exist at a specifically high expression level in stages 5, 1, 3, and 2, respectively. LncRNAs in cluster 5 were higher expressed during the non-thermogenic period of the receptacle (stages 1 and 5) (Figure 2D). The significant stage-specific expression patterns indicated that these DELs were subject to different degrees of transcriptional regulations and played a vital role during specific periods.

### 3.2. The Identification of Floral Thermogenesis-Related lncRNAs by WGCNA

WGCNA is the most commonly used system biology method to date, which can be used to identify co-expressed gene modules and analyze the correlation between the modules and phenotypic data [37]. In this study, WGCNA was performed on all transcripts to screen out the floral thermogenesis-related modules. The MEpink and MEpurple modules were positively correlated with thermogenic traits by calculating the correlation between the gene modules and phenotypes (Figure 5B). The expression levels of MEpink and MEpurple modules in thermogenic phase (stages 2–4) were higher than that of non-thermogenic phase (stages 1 and 5) (Figure S1). The gene expression pattern in these two modules was consistent with the trend of receptacle temperature change. Hub genes of these two modules were also identified to further understand the co-expression network. The results showed that *AOX1a* (NNU_06050) and *AOX1b* (NNU_06051), as floral thermogenesis related genes in *N. nucifera*, were identified as HUB genes of MEpurple and MEpink module, respectively [35]. At the same time, HUB genes were also the included genes involved in redox activity and the pentose phosphate pathway, such as *PGL2*, *LPD1*, and *CYP77A3*. *PGL2* can catalyze the hydrolysis of 6-phosphogluconolactone into 6-phosphogluconate. The pentose phosphate pathway can not only provide raw materials for anabolism, but can also provide energy for organisms [38]. Therefore, the transcripts in the MEpink and MEpurple modules are closely related to floral thermogenesis.

In order to reveal the function of the MEpink and MEpirple modules, GO-enrichment analysis was conducted on them in the present study. The results show that sulfate absorption is the most significantly enriched GO term in the MEpurple module (Figure S2). Sulfur (S) is an important component of amino acids, chloroplasts, vitamins. and coenzymes, which is essential for plant growth. Therefore, S plays an important role in photosynthesis, respiration, and the formation of the cell membrane structure in plants [39,40,41,42]. The MEpink module is enriched in terms of “electron transport coupled by mitochondrial ATP synthesis”. Moreover, the GO-enrichment analysis indicates that genes in the MEpink module are also enriched in terms of “electron transport, ubiquitin to cytochrome C”, and plays a negative regulation role in this pathway (Figure S3). Previous studies have shown that alternative respiratory pathways may be enhanced when the cytochrome pathway is inhibited [43]. In the plant mitochondrial respiratory chain, electrons are shunted from quinone and directly transferred to molecular oxygen through alternating oxidase, bypassing phosphorylation site III. The synthesis of ATP is decreased and a large amount of energy is released as heat. Then, plants begin to produce heat autonomously [27,35]. Therefore, these results indicate that lncRNAs in the MEpurple and MEpink modules might be involved in floral thermogenesis by regulating the genes associated with cellular respiration and the energy metabolism pathway.

### 3.3. The Function of lncRNA as eTMs for miRNA

LncRNAs can regulate gene expression through various complex mechanisms. In the gene post-transcriptional regulation level, lncRNA can act as bait to prevent miRNA from binding to target genes [44]. In *Arabidopsis*, eTMs of miR160 and miR166 have been validated as functional target mimics and their roles in controlling plant development have been determined [45]. In the present study, 70 DELs could compete with 16 floral thermogenesis-related mRNAs to bind 29 known miRNAs (Table S4). Many temperature-response miRNA families were involved in these 29 miRNAs, such as miR160, miR164, miR167, miR169, miR168, miR319, and miR393. For example, the expression levels of miR319, miR393, miR168, and miR169 were increased by 1.5 times after low-temperature treatment in *Arabidopsis thaliana* [46]. Meanwhile, the role of miR319 in regulating low-temperature stress tolerance has been studied in rice. Overexpressing miR319 had an increased survival rate, compared to WT plants under cold stress [47,48]. In the previous study, these temperature-responsive miRNAs were also differentially expressed between floral thermogenesis stages and non-thermogenesis stages. For example, the average expression level of miR160a-5p in the thermogenic period was about 40 times that of the non-thermogenic period. However, the average expression level of miR398b in the non-thermogenic period was about 10 times higher than that in the thermogenic period [28]. Therefore, the co-expression network of lncRNA–miRNA–mRNA may play a role in the thermoregulation of lotus. The dual-luciferase reporter assay was used to verify the interaction of LT_00068702-MIR164a_4-NNU_11990 in the protoplasts of lotus. The NNU_11990 gene encodes thioredoxin-like protein *TrxL2*, which is the oxidizing factor of redox-regulatory proteins [49]. Redox status determination and enzyme activity determination assays have shown that the *TrxL2* was involved in the oxidative activation of glucose-6 phosphate dehydrogenase (G6PDH), which is the first step in the pathway to catalyze the oxidation of the pentose phosphate pathway (OPPP) [50]. At the same time, thioredoxin can also regulate chloroplast ATP synthase [51]. Overall, *TrxL2* (NNU_11990) plays an important role in the energy metabolism of cells. Therefore, LT_00068702 might act as the eTMs for miR164a_4 to regulate the expression of *TrxL* (NNU_11990) and participate in the floral thermogenesis regulation of *N. nucifera*.

## 4. Materials and Methods

### 4.1. Plant Materials

The cultivar used in this study was “Mantianxing”, which was planted in the Wuhan National Germplasm Repository for Aquatic Vegetables (30°12′ N, 111°20′ E), in Wuhan City, Hubei Province, China, and its deposition number was V11A0692. According to Grant, the flower of *N. nucifera* can be divided into five distinct developmental stages [35]. The receptacles of these five stages were collected, immediately frozen in liquid nitrogen, and stored at −80 °C.

### 4.2. Strand-Specific RNA Sequencing

The total RNA was extracted from the receptacles of each stage using pBIOZOL (BIOER, Hangzhou, China) reagent, according to the manufacturer’s instructions. Agilent 2100 BioAnalyzer (Agilent, Santa Clara, CA, USA) was used to detect the quality of the total RNA. The samples that met the sequencing quality standards could be used for subsequent library construction and sequencing. A total of 15 cDNA libraries were constructed with 3 biological replicates for each stage of the receptacle. The experiment pipeline steps for strand-specific sequencing were as follows: Ribo-Zero™ rRNA Removal Kit (Illumina, San Diego, CA, USA) was used to remove the ribosomal rRNA from the total RNA. according to the instructions. After RNA fragmentation, TruSeq^®^ Stranded kit (Illumina, San Diego, CA, USA) was used to synthesize cDNA, according to the manufacturer’s instructions. Subsequently, the “A” base and adaptor were ligated to the double-stranded cDNA products and PCR was performed to amplify the products. The final cDNA libraries were obtained after purification. Finally, the constructed sequencing libraries were sequenced on the Illumina HiSeq platform.

### 4.3. The Identification of lncRNA

Clean reads were obtained by several filtering steps, including removing rRNA, low-quality reads, adaptor-contaminated reads, and high N-base reads. Clean reads were mapped to the *N. nucifera* reference genome by HISAT software [52]. Subsequently, transcripts were assembled using Stringtie and CuffMerge software [53,54]. Four methods were used to predict the coding ability of the assembled new transcripts, including three types of software (CPC_threshold, CNCI_threshold, and txCdsPredict_threshold) and the pfam protein database. The scoring threshold of CPC_threshold and CNCI_threshold software was 0, while that of the txCds Predict_threshold software was 500 [55,56,57]. The transcripts with coding ability scores below the threshold were predicted as lncRNAs. Only when at least three of the four methods were consistent, did we confirm that the transcript was mRNA or lncRNA.

### 4.4. Screening of the DELs and the Analysis of Their Target Genes

The differential expression analysis of lncRNAs were performed between different developmental stages by DEGseq software [58]. The filtration condition was fold change ≥ 2.00 and adjusted *p*-value ≤ 0.001. The pipeline steps for the target gene prediction were as follows: Spearman and Pearson correlation coefficients of lncRNA and mRNA were calculated, the filtration condition was Spearman _COR ≥ 0.6 and Pearson _COR ≥ 0.6. Then, the lncRNA located within 10 kb upstream or 20 kb downstream of protein-coding genes on the same chromosome were searched as cis-acting mode and the adjacent mRNA was screened out as its target gene. Trans targets were determined by Weighted Gene co-expression Network Analysis (WGCNA), independent of location relationship http://www.ehbio.com/Cloud_Platform/front/ (accessed on 1 July 2020). The target genes of lncRNA were annotated using BLAST or Diamond, according to the NT, NR, KOG, KEGG, and SWISSPROT databases [59,60]. GO annotation was performed using Blast2Go [61]. The GO-enrichment analysis of the target gene of lncRNA was performed by agriGO [62].

### 4.5. Quantitative Real-Time PCR Validation

The Revertaid^TM^ First Strand cDNA Synthesis Kit (Thermo, MA, USA) was used to reverse transcribed 1 µg total RNA into cDNA. Then, the cDNA products were diluted 10 times for the qRT-PCR assay. A FastStart Universal SYBR Green Master (ROX) kit (Roche, Basel, Switzerland) was used to detect the expression levels of lncRNAs on an ABI StepOnePlus Real-Time PCR System. The reaction procedure was as follows: 95 °C for 10 min; then 40 cycles of 95 °C, 15 s, 60 °C, 1 min. *Actin* gene of *N. nucifera* was used as the endogenous reference gene. The results were calculated by the 2^−ΔΔCT^ method, and each experiment was repeated three times [63]. GraphPad Prism 7 software was used for the statistical analysis of the qRT-PCR data. All the primer sequences of qRT-PCR are listed in Table S6.

### 4.6. The Construction of the ceRNA Network

The miRanda algorithm was used to predict the interaction between miRNA and the target gene [64]. For ceRNAs pairs with no less than three shared miRNAs, the hypergeometric distribution was used to calculate the significance of shared miRNAs, and the false discovery rate (FDR) was used to correct the *p*-value. The gene pairs with FDR ≤ 0.05 were selected for downstream analysis. Cytoscape software (version 3.5.0) (accessed on 1 May 2021) was used to visualize the ceRNA networks.

### 4.7. Lotus Protoplast Preparation and Dual-Luciferase Reporter Assay

Plant protoplast preparation and transformation kits (Real times, Beijing, China) were used to isolate the protoplasts of lotus, according to the manufacturer’s instructions. Subsequently, 100 µL of protoplast solution were co-transfected with 10 µg of plasmids of lncRNA, luciferase, and miRNA. LTCONS_00068702 and the miR164a_4 precursors were cloned into the pCXUN vector with a ubiquitin promoter, respectively. The target mRNA NNU_11990 was inserted into the dual-luciferase reporter vector pGreenII 0800-LUC. The transfected protoplasts were incubated at 28 °C for 18 h to express RNA. The dual-luciferase reporter assay was performed using the Firefly & Renilla Dual-Luciferase Assay Kit (US Everbright^®^ Inc., California, USA). GloMax 20/20 Luminometer (Promega, Madison, WI, USA) was used to measure the luciferase activity. Renilla Reniformis luciferase was used as the endogenous reference gene. All primer sequences of gene cloning are listed in Table S7.

## 5. Conclusions

In the present study, we reported the genome-wide identification of lncRNAs related to floral thermogenesis in *N. nucifera* through high-throughput strand-specific RNA sequencing. A total of 22,693 transcripts were identified as lncRNAs from receptacles at five different developmental stages, of which approximately 44.78% had stage-specific expression patterns. Among them, more lncRNAs showed stage-specific expressions at non-thermogenesis periods (stages 1 and 5) than those in the thermogenesis periods (stages 2, 3, and 4). Subsequently, we identified 2579 DELs regulating 2367 protein-coding genes, mainly involved in receptacle development and the reproductive process.Then, lncRNAs with floral thermogenesis identified by WGCNA were mainly related to sulfur metabolism and mitochondrial electron transport chains. Meanwhile, 70 lncRNAs were predicted to act as endogenous target mimics for 29 miRNAs and participate in the regulation of 16 floral thermogenesis-related genes. Our dual-luciferase reporter assays indicated that lncRNA LTCONS_00068702 acted as eTMs for miR164a_4 to regulate the expression of the *TrxL2* gene. In short, lncRNAs may play important roles in floral thermogenesis by noncoding regulators on their target genes or acting as miRNA sponges, which will further enrich the research on ncRNAs in *N. nucifera* and provide candidate lncRNAs for future studies.

## Data Availability

All the sequencing clean data of this study are available in the NCBI Sequence Read Archive with the accession number PRJNA548651.

## Supplementary Materials

The following supporting information can be downloaded at: https://www.mdpi.com/article/10.3390/ijms23094901/s1.
