# Supplementary material for: The Genome-Wide Identification of Long Non-Coding RNAs Involved in Floral Thermogenesis in Nelumbo nucifera Gaertn
Source: Int J Mol Sci. 2022 Apr 28;23(9):4901. doi: 10.3390/ijms23094901 (PMC9102460; doi:10.3390/ijms23094901)
Supplement: Supplementary file 1 [file ijms-23-04901-s001.zip › Table S1.pdf]

Table S1 Mapping ratio statistics of cleanreads with genome in ssRNA-sequencing

Samples	Raw reads	Clean reads	CleanReads Ratio	Unique Mapping Ratio	Mapped ratio
Stage 1_1	127,366,118	126,210,332	99.093%	64.08%	65.36%
Stage 1_2	130,607,632	126,620,672	96.947%	67.91%	69.18%
Stage 1_3	130,618,136	126,619,886	96.939%	66.48%	67.74%
Stage 2_1	128,984,692	125,836,884	97.560%	65.06%	66.37%
Stage 2_2	130,617,568	126,324,178	96.713%	66.47%	67.83%
Stage 2_3	129,030,574	126,165,640	97.780%	67.95%	69.23%
Stage 3_1	130,606,984	126,319,706	96.717%	67.96%	69.46%
Stage 3_2	130,610,690	125,936,890	96.422%	65.91%	67.50%
Stage 3_3	130,609,062	127,340,280	97.497%	66.16%	67.59%
Stage 4_1	130,613,690	127,138,946	97.340%	67.43%	68.76%
Stage 4_2	130,610,100	125,949,118	96.431%	69.61%	71.06%
Stage 4_3	130,607,846	126,647,466	96.968%	64.14%	65.66%
Stage 5_1	130,607,096	126,784,988	97.074%	64.81%	66.21%
Stage 5_2	131,834,936	126,575,834	96.011%	62.58%	64.22%
Stage 5_3	123,565,310	119,055,696	96.350%	65.04%	66.40%
