# Supplementary material for: The Genome-Wide Identification of Long Non-Coding RNAs Involved in Floral Thermogenesis in Nelumbo nucifera Gaertn
Source: Int J Mol Sci. 2022 Apr 28;23(9):4901. doi: 10.3390/ijms23094901 (PMC9102460; doi:10.3390/ijms23094901)
Supplement: Supplementary file 1 [file ijms-1676208 -supple fig proofed.pdf › ijms-1676208 -supple fig proofed.pdf]

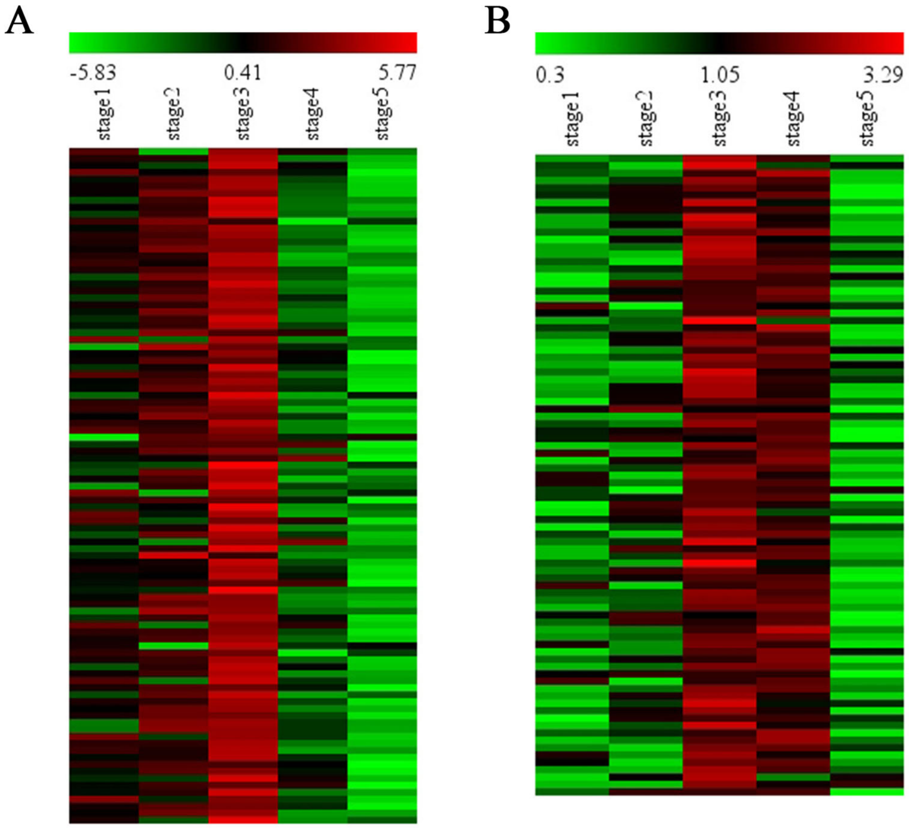

Figure S1. Heatmap of transcript expression in the MEpink and MEpurple modules. (A) Expression patterns of transcripts in the MEpink module. (B) Expression patterns of transcripts in the MEpurple module.

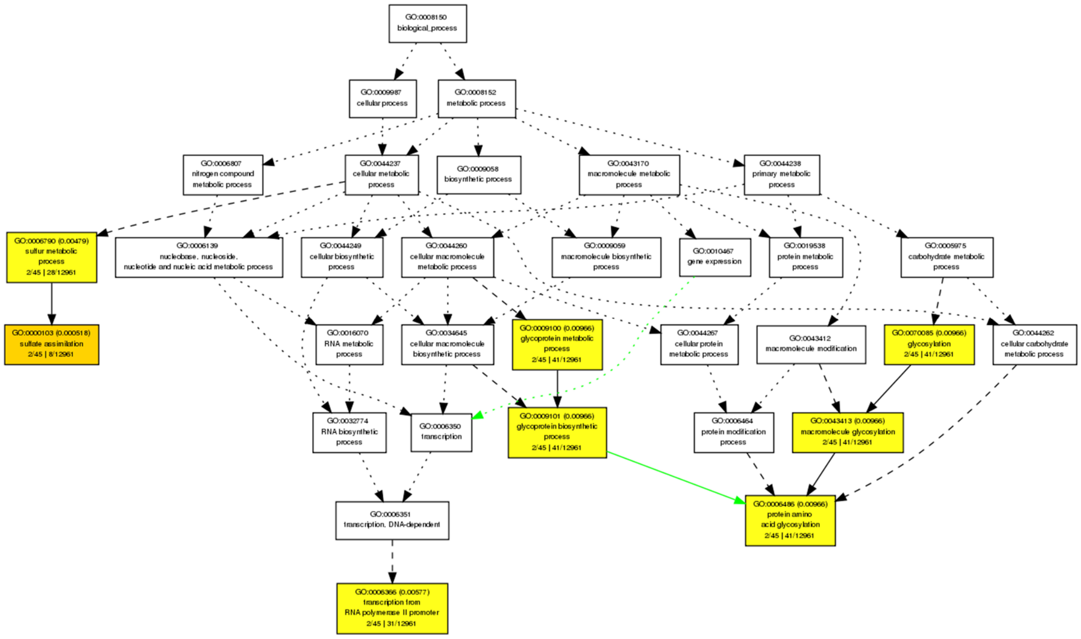

Figure S2. GO-enrichment analysis of the genes in the MEpurple module.

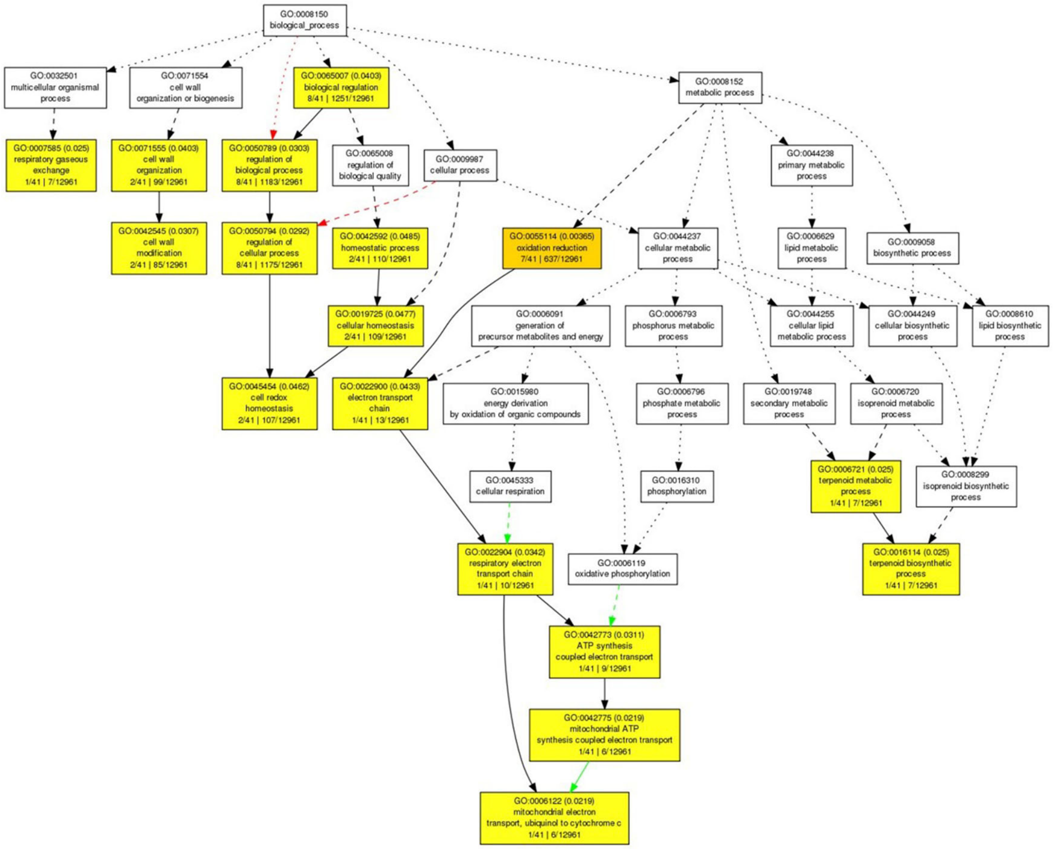

Figure S3. GO-enrichment analysis of the genes in the MEpink module.
